# Impact of the Case Management Model through Community Liaison Nurses

**DOI:** 10.3390/ijerph16111894

**Published:** 2019-05-29

**Authors:** Gonzalo Duarte-Climents, María Begoña Sánchez-Gómez, José Ángel Rodríguez-Gómez, Cristobalina Rodríguez-Álvarez, Antonio Sierra-López, Armando Aguirre-Jaime, Juan Gómez-Salgado

**Affiliations:** 1 University School of Nursing, Candelaria NS University Hospital, University of La Laguna, Canary Islands Health Service, 38010 Santa Cruz de Tenerife, Spain; extgduartcl@ull.edu.es; 2 University School of Nursing and Physiotherapy, Health Sciences School, University of La Laguna, 38200 Santa Cruz de Tenerife, Spain; jarogo@ull.es; 3 Department of Preventive Medicine and Public Health, University of La Laguna, 38200 Santa Cruz de Tenerife, Spain; crrodrig@ull.edu.es (C.R.-Á.); asierrasoprania@hotmail.com (A.S.-L.); 4 Research Support Unit for Primary Care Management and Candelaria NS University Hospital, 38010 Santa Cruz de Tenerife, Spain; armagujai@gmail.com; 5 Department of Sociology, Social Work and Public Health, University of Huelva, 21007 Huelva, Spain; salgado@uhu.es; 6 Safety and Health Posgrade Program, Universidad Espíritu Santo, Guayaquil 091650, Ecuador

**Keywords:** primary health care, community nursing, case management, polypathological patients, quality of health care

## Abstract

The objective of the present study is to assess the model’s impact on patients and their families in terms of outcomes and the efficiency results for the health system in Tenerife, Canary Islands, selecting a period of eight years from the time interval 2002–2018. The employed indicators were collected on a monthly basis. They referred to home care and its impact on clinical outcomes and on the use of resources. The comparison between the indicators’ tendencies with and without the liaison nurse model was done with the F-test by Snedecor. All these tests are bilateral, with a level of significance of *p* < 0.05. In those areas with community liaison nurse (CLN), improvements have been found in indicators that describe: (1) the management of the clinical status of patients, (2) the efficiency of the use of resources, and (3) the quality and compliance with the process that also includes home visits and social risk detection and management. It can be said that in the basic areas of primary health care where the work of the CLN develops there are improvements in the management of the patients’ clinical condition as well as in the quality and efficiency of care.

## 1. Introduction

Case management is a polysemous term. The King’s Fund report of 2011 [1] makes use of the definition given by Hutt in 2004 as “the process of planning, coordinating, and reviewing the care of an individual” [2], and completes it with the definition of the Case Management Society of America: “a collaborative process of assessment, planning, facilitation, care coordination, evaluation, and advocacy for options and services to meet an individual’s and family’s comprehensive health needs through communication and available resources to promote quality cost-effective outcomes” [3].

The first publication appearing in Medline on the case management topic dates back to the year 1878 and refers to the management of a patient with cerebral empyema [4]. When narrowing the search to the field of nursing, we find an article from 1979 that covers the management of psychiatric patients through nurse case managers [5], followed by another study from 1984 on the area of geriatrics [6]. The search for systematic reviews brings back a study from 1983 which provides case management as a strategy for an intervention on childhood diarrhoea [7]. The first systematic review of the impact coming from the results of different case management strategies with nurses in primary care was given by Ferguson in 1998 [8].

The concept of case management, in general, deals with the process of care both to patients and relatives, the complexity of problems, coordination between professionals, and the intervention. Certain disagreement is found regarding this last aspect on whether it is punctual or continuous in time, although, in general, it refers to continued care [9,10,11]. We understand that managing cases in nursing means assessing the care needs of a patient and his/her environment and coordinating the contribution of different health care providers. Case management is a response directed to improving the continuity of care for particularly polypathological patients or those who require the contribution of different care providers to meet their needs [12,13,14,15].

Nurse case managers’ target patients are people with a high need for care associated with health problems, polypathologies, mostly but not necessarily chronic comorbidities, or terminal processes, which are linked to situations of dependency. These situations have a great impact on the social and family environment of these people and, in particular, on their caregivers. The volume of this type of patient increases with the aging process of the population as well as with situations of disability and the changes in the family model that come along with a reduction of available support [16].

Caring for these complex patients involves different services areas, different levels of care, and different professionals. The involved services areas include, at least, the health and social welfare sectors, levels from primary care to attention from specialists, and all the involved professionals, that is, auxiliary nurses, nurses, physiotherapists, midwives, social workers, psychologists, and physicians. In this complex scenario, the coordination of all the resources that are necessary for the continuity of care in these cases requires the identification and development of services towards obtaining maximum effectiveness and efficiency [17].

In the Canary Islands, the nurse case manager has been called “community liaison nurse” (CLN) and was introduced, prior assessment of its impact by the Canary Islands Health Service, in 2000 as a proposal for the improvement of home care based on the case management approach [12,18]. Since then, the figure of the community liaison nurse has widened and been implemented in the rest of Spain during the 2010s in different care contexts and health services [13].

The present study meets the need to assess the impact of the community liaison nurse model for case management after having completed the training period and being operating in a stable way. The hypothesis is that this model has a positive impact on the control of the patients and their families’ state of health and produces efficiency improvements for the health system. Consistent with this hypothesis, the objective was to assess the impact of the model in terms of outcomes for the patient and his/her family and the efficiency results for the health system in Tenerife, Canary Islands. The study was developed during the period 2002–2018, selecting a period of years that was not affected by, on the one hand, the implementation of the model and, on the other hand, the adjustment of resources provoked by the crisis.

## 2. Materials and Methods

The study took place in Primary Care Centres of Tenerife, Canary Islands, Spain, during the period 2002–2018. In 2002, the Canary Islands Health Service included the model in its services portfolio. Of this period of 16 years, a group of 8 years was finally selected for data extraction, 2004–2012 because, on the one hand, it was when the community liaison nurse model’s stability phase was reached (2002–2004) and, on the other hand, the years most severely affected by the crisis were eliminated (2013–2018). This period was chosen as it was when the community liaison nurse model’s operating stability phase had been reached. The information used in the study belongs to those centres that either had the service, that is, the intervention group, or did not have the service, that is, the control group, during the studied period. Those centres in transition from the previous model to the community liaison nurse were excluded so as to avoid indicator bias subject to the influence of the learning curve.

The indicators used have a monthly periodicity and are referred to home care and its impact on clinical outcomes and use of resources. These indicators are available both for the basic health areas with liaison nurses and for those that have not integrated this figure. They are divided into activity or process indicators (1 to 5) and result indicators (6 to 9):

1. Home care coverage: Relationship between home care persons included and expected (14% of those older than 65 years included). Terminal and immobilised patients aged less than 65 were included in the numerator.
2. Coverage of caregivers for continuity care related to the confined: Percentage of caregivers regarding confined persons included in the Home Care Continuity Care Service.
3. Coverage of workshops for caregivers: Percentage of caregivers included in the caregivers’ workshop.
4. Confined with more than four visits to the Family Care Unit: Percentage of confined persons within the home care service who have had four or more home visits from physicians or nurses who are not liaison nurses in the last year.
5. Social risk assessed by the social worker: Percentage of persons in social risk included in the Home Care Continuity Care Service who have been assessed by social workers.
6. Hospitalisations: Percentage of hospital admissions in the last year among the people included in the quota as confined within the Home Care Continuity Care Service.
7. Pressure ulcers: Percentage of patients with pressure ulcers among the people included in the quota as confined within the Home Care Continuity Care Service.
8. Falls: Percentage of patients with falls among the people included in the quota as confined within the Home Care Continuity Care Service.
9. Excess effort of caregivers: Percentage of patients with caregiver’s excess effort among the people included in the quota as caregivers in the Home Care Continuity Care Service.

### Data Processing

With the monthly values sequences of each indicator, two time series were formed. One for the group of centres which had integrated the community liaison nurse model into their service portfolio and the other for the group of centres without this model. Each series consisted of the indicator values mean for each group of centres and each month of the period. The secular tendency of the series thus shaped was estimated. For the estimation of the tendency, the adjustment of several models to the series was used, selecting the most suitable one by their root-mean-square values and their residual behaviour. It was verified that the residues behave as random sequences close to a standardised normal distribution with mean 0 and deviation 1. The presence of oscillating patterns in the residues was explored by periodic movements in the high frequencies or short periods. If these patterns were detected, series’ straightening methods of the mobile mean type of 2–3 points or exponential were used in order to eliminate them. The statistical significance of the tendency models’ coefficients that indicate the way of progression of the series was obtained. The comparison between the indicators’ tendencies with and without the liaison nurse model was carried out with the Snedecor’s F-test. All these tests are bilateral with a *p* < 0.05 level of significance. Calculations were made with the help of the Statistical Package for the Social Sciences (SPSS version 21.0, SPSS Inc., Chicago, IL, USA).

## 3. Results

**Figure 1:**
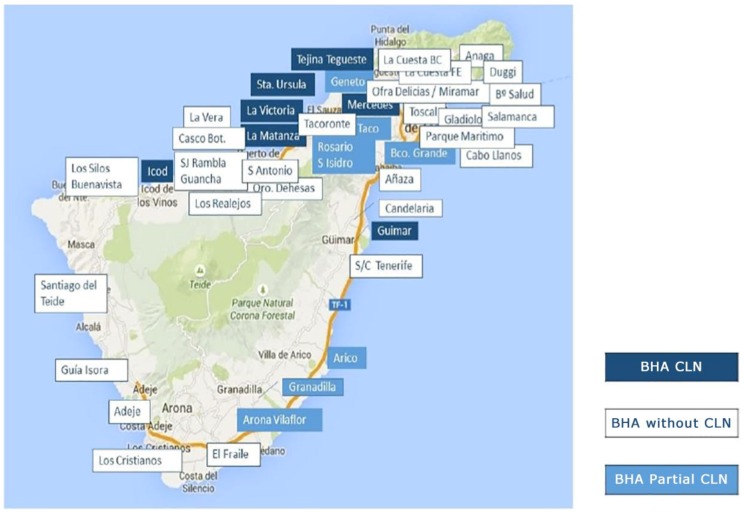
Distribution of health basic areas in Tenerife with and without community liaison nurse.

The geographical location of the basic health areas that compose the intervention group with the CLN model and those that compose the control group, i.e., health centres without this model, are shown in Figure 1. This figure also shows the areas where the CLN has been implemented during the study period and those that have been excluded from the analysis. Apart from their geographical location, the health areas do not differ substantially in terms of coverage, type of assisted population, and local socio-economic level, as the zoning itself considers the homogeneity of these characteristics.

**Figure 2:**
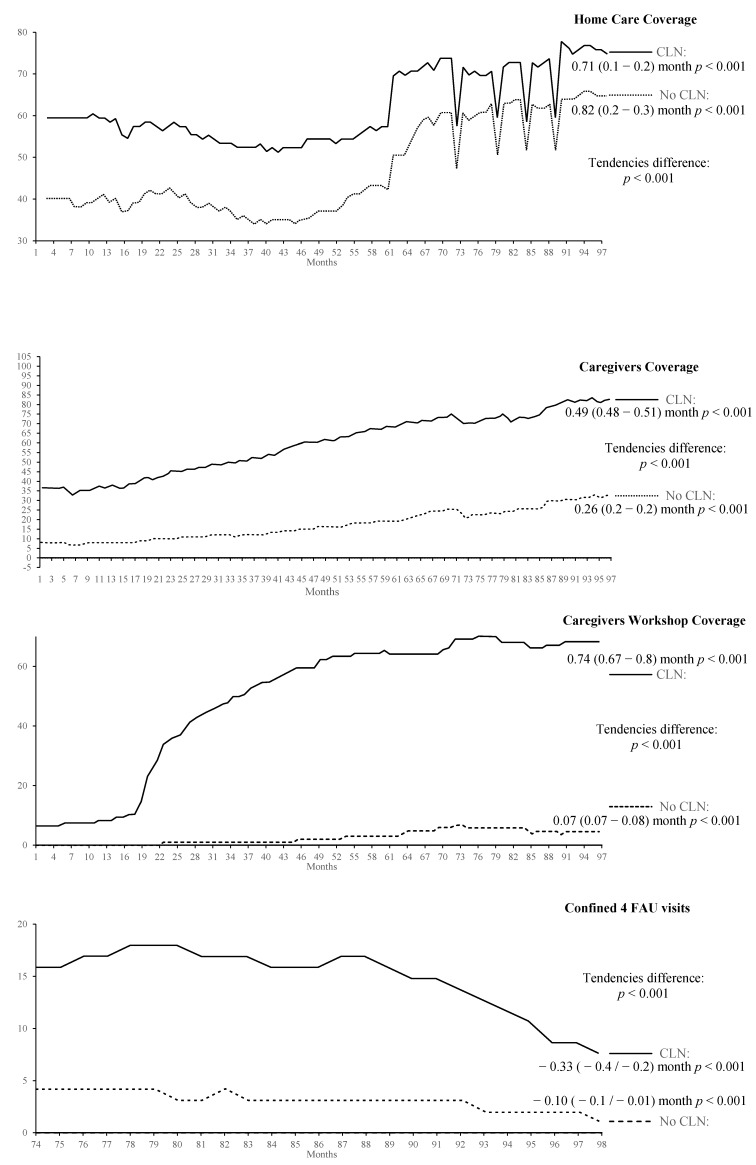
Estimation outcomes of the process indicators, tendency, and difference signification.

**Figure 3:**
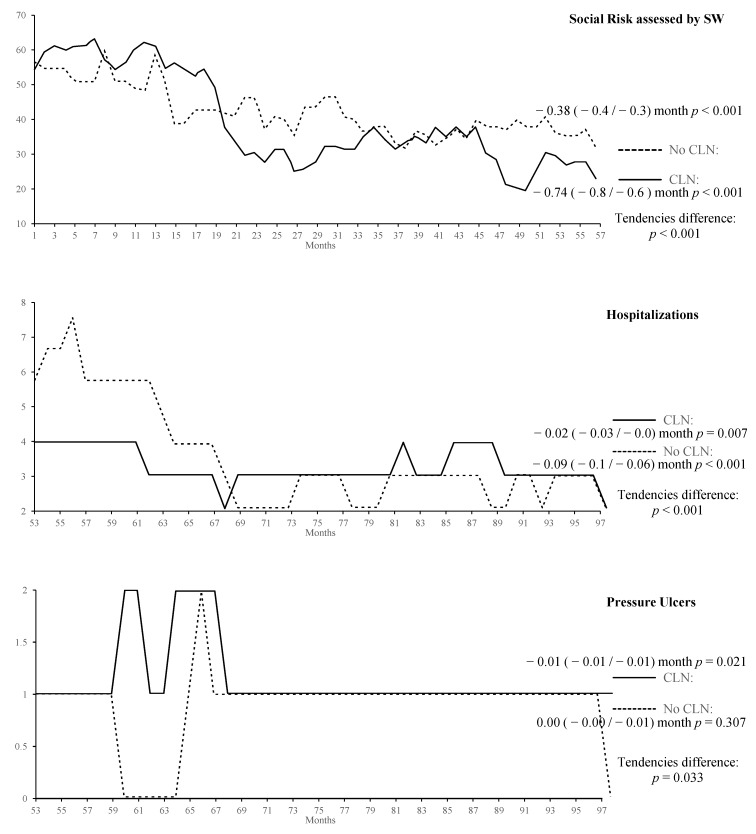

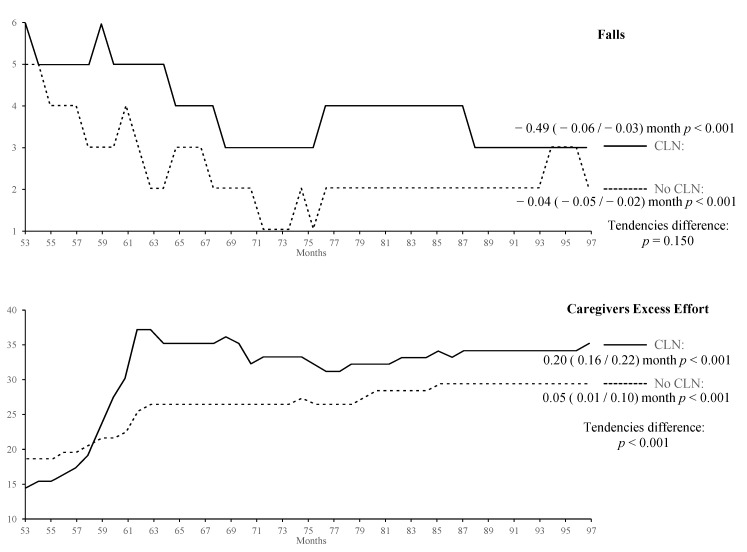
Estimation outcomes of the patients’ outcome indicators, tendency, and difference signification.

The graphical form of the indicators’ progression, the results of their tendency analysis, and the significance of the tendency differences between basic health areas with and without CLN are shown in Figure 2 and Figure 3, grouping the process indicators in Figure 2 and those of patients’ results in Figure 3.

As shown in the charts in Figure 2, the home care coverage for all health areas that have CLN starts at a mean level of 60% while for those who do not have the CLN starts at 40%. The home coverage progression for the CLN shows a growing tendency with a monthly rate of 0.71 (CI 95%: 0.16–0.25) while in the case of the traditional nurse this tendency shows a slight decrease of 0.01 (CI 95%: 0.9–0.11). The difference between these tendencies reaches statistical significance. The graphs of the continuity care caregivers coverage, the workshops for caregivers, and those corresponding to the confined with more than four Family Assistance Unit visits show similar tendencies, with more pronounced differences in the basic health area group with CLN. Statistical significance is also found in the tendencies’ differences. The fifth indicator of this group is the social risk valued by the social worker. In this indicator, the graphic differences are not as pronounced as in previous indicators. Again, a statistical significance for the tendencies’ differences is found in favour of the CLN group. For these last two indicators, the data availability did not allow to complete the series. For the indicator “Family Assistance Unit visits”, a series of 24 months is considered and for the social risk the series is of 57 months.

Figure 3 shows the graphs for indicators related to outcome indicators in patients. In the four indicators, we find a temporary series that goes from month 53 to 98. Hospitalisations, pressure ulcers, and caregiver excess effort show significant tendencies’ differences in all three cases in favour of the liaison group. These differences are displayed in the graphs. Although, regarding falls better results are also observed in favour of the CLN group, the difference does not reach a statistical significance.

## 4. Discussion

The results of this study show that in the basic health areas where the work of community liaison nurses is carried out improvements are obtained in indicators that describe, (1) the management of the clinical state of patients such as hospital admissions, pressure ulcers, and the detection of family caregivers’ excess effort; (2) efficiency in the use of resources such as hospitalisations, home visits by the Family Assistance Unit, and social risk detected by the liaison nurse and assessed by the social worker; and (3) quality and compliance with the process including coverage for patients in home care and that of their caregivers and the coverage of the specific service of workshops for caregivers, among which home visits and social risk detection and management are also included.

When comparing our results with those published by other investigators, we found a serious limitation in the reviews, that is, the absence of meta-analysis of the results due to the methodological heterogeneity, the profiles of patients described, and the indicators used in the different studies available. However, there are systematic reviews on case management systems that can be approximated to our assessed model. Thus, the revisions by Doughty [19], Chiu et al. [20], Oeseburg et al. [21], You et al. [22], and Trivedi et al. [23] share the same target population, i.e., frail elderly people. The study by Keleher et al. [24] coincides in the area of primary care. The rest of the works mix the areas of primary care and hospital care [25,26,27,28,29,30,31,32,33,34,35,36,37,38,39]. Several revisions agree in the decrease in hospital admissions, readmissions, and prevention of admissions through case management. In a concrete way, this translates into less hospital admissions for elderly people (Chiu et al. [20]), fewer readmissions for people with heart failure (Slyer et al. [31]), less admissions in children with chronic processes (Coller et al. [35]), and less admissions for different profiles of patients in Joo’s work [37]. Holland et al. also found that cross-sector community health teams made a more limited use of acute care services such as hospitalisations with subsequent reduction of health care expenditure [40].

Although the results found through the time series indicators of Tenerife and the literature review go in the same direction, when comparing both outcomes, we encounter the difficulty of finding an equal model to the assessed. There is a great diversity of services provided through case management. We must keep in mind that the service provided by liaison nurses in the Canary Islands is the first of these characteristics within the primary care area in Spain. Case management applied to the improvement of home care was the assessed model in this study while in the literature there are similar models applied to mental health (depression), chronic processes (diabetes, obstructive pulmonary disease), the elderly, and children. The specific indicators of each study are also different although most of the results are in the same positive direction. In general, we can conclude that the results found in our study are consistent with what was published by other authors in different contexts and for different patient profiles [39].

Our study service focuses on people included in the Home Care Programme of the Canarian Health Service and their caregivers, mostly elderly, dependent, polypathological, polymedicated and with female caregivers who also are their relatives and, most of them, polypathological and polymedicated as well. This profile of target population is similar to the one studied by Morales-Asencio et al., the results of which revealed that case management interventions, due to the complexity of the patients assisted, led to implementing behavioural interventions, navigation through the health system, and clinical safety [41].

Available studies show the case management model applied to broader profiles of people in need of care: elderly, dependent, and with chronic and mental health problems. The target populations, elderly who require home care in our case and elderly, polypathological, with mental health problems and chronic processes both in adults and children in other studies, allow framing them all within the most complex identified population group in the stratification models. According to the Kaiser pyramid model, 5% of the population is in this group, with a consumption of 60% of the health resources [42,43]. Nurse case managers are helpful in identifying the personalised necessary care for the patient and in facilitating the process of care, being an added value for the patients [44] and their family health [45].

The improvements obtained in the health indicators of this study are in line with the perception of the interprofessional healthcare team members. According to Powell et al., they identified the barriers of the health care system by addressing the patients’ needs. These included poor care coordination, inadequate communication of hospital discharge instructions, and difficult and complex navigating systems. In order to face these barriers, some actions were suggested including enhanced communication between care sites, which is the purpose of the CLN [46]. The authors also suggested a patient-centred scheduling, which is associated with a growth in use of case manager staff [47]. The involvement of community members in the health care system is a field in need of development for advanced nursing practice, as described in the People-Centered Care Partnership Model. This model aims at building new values concerning health and forming a social system that guarantees quality of life and social support. In this context, the CLN could play an essential role in building partnership between the health system and the communities [48].

Our study is affected by some limitations and biases. The first limitation is having based the analysis on monthly elaborated indicators from the information that professionals introduce daily in the clinical records. This indicates that the process does not have any quality control system, nor is governed by a reporting discipline of regulated notifications, so we can assume an insufficient quality of data. Therefore, our study could be affected by an underestimation bias of the impact of the CLN model on the used indicators, so we assume that any improvement in the quality of data record in the clinical records would improve the results presented here. A second limitation is having employed in the analysis the available indicators designed and used by the General Directorate of Care Programme. Among these, it is evident the absence of specific indicators which differentiate, for example, concepts such as “hospitalisations” and “readmissions”, or those concepts related to the nurses’ work which distinguish, for example, in an indicator such as “at least four visits of the Family Assistance Unit”, which professional visit is needed. The absence of such indicators impedes a deeper analysis of the impact of the CLN model. A third limitation is the lack of disaggregation of the indicators aimed at the assessment of macromanagement, mesomanagement, and micromanagement, which would have been very useful when assessing the CLN model outcomes at these levels. In this sense, the taxonomy proposed by the American College of Cardiology [49] is a step forward towards classifying the different case management services that can be expanded towards key indicators that facilitate assessment and benchmarking. A fourth limitation is having restricted the study to the island of Tenerife, although we believe the results of our study can be extrapolated at least to the rest of the territory of the Canary Islands as the CLN model shares the same target population in the rest of the islands and the same working protocol. Beyond the autonomous community, the results could be extrapolated to the extent that the target population and case management protocol are similar to those employed by the CLN in the Canary Islands. The model is expected to succeed in the nationwide Spanish context after having analysed the benefits of the case manager as a starting point for the advanced practice nursing implementation. Sánchez-Gómez et al. identified the positive impact on patients in terms of health results, satisfaction, and life quality as the advanced practice nurse performs a more effective follow-up of chronic patients with a better control of risk factors, symptoms, and health outcomes and an earlier detection of complications [50].

## 5. Conclusions

Taking into consideration the limitations and biases that affect our study, we can affirm from its results that in the basic areas of primary health care where the work of the CLN develops improvements are obtained in the control of the clinical state of patients, in quality and efficiency of care. However, more studies are required using more specific and controlled indicators to confirm the conclusions derived from our study’s outcomes.

A strength of this study is that we identified the improvements achieved by the case management model and the CLN in the clinical state of patients, in the efficiency in the use of resources, and in the quality and compliance with the process. As weaknesses of the study, we identified the data sources, the standard indicators, the lack of disaggregation in different types of case management, and the local population. In order to deal with these issues, we suggest rigorous quality research with a more controlled method of data recording, the use of specific indicators of the expected outcomes, a clear case management service classification, and a wider study population.

Case management is an open path for a better use of health resources and a cost-effective health service. CLN is an extended nursing role that allows nurses to provide a wider service to the community, higher quality, and personalised care.
